# Isolation and Characterization of a New Ginsenoside from the Fresh Root of *Panax Ginseng*

**DOI:** 10.3390/molecules15042319

**Published:** 2010-03-30

**Authors:** Chang-Chun Ruan, Zhi Liu, Xiang Li, Xia Liu, Li-Juan Wang, Hong-Yu Pan, Yi-Nan Zheng, Guang-Zhi Sun, Yan-Sheng Zhang, Lian-Xue Zhang

**Affiliations:** 1 Institute of Agricultural Modernization, Jilin Agricultural University, Changchun, 130118, China; E-Mails: ccyuan@yahoo.com (C.C.R.); lzhiiu@126.com (Z.L.); gzsun1967@126.com (G.Z.S.); 2 College of Chinese Medicinal Materials, Jilin Agricultural University, Changchun, 130118, China; E-Mails: ljwang@yahoo.com (L.J.W.); zhenyinan@tom.com (Y.N.Z.); 3 Agriculture and Agri-Food Canada, Saskatoon Research Center, 107 Science Place, Saskatoon, S7N 0X2, SK, Canada; E-Mail: drxiang@hotmail.com (X.L.); 4 College of Plant Science, Jilin University, Changchun, 130062, China; E-Mail: panhongyu@jlu.edu.cn (H.Y.P.); 5 Wuhan Botanical Garden, Chinese Academy of Sciences, Wuhan, 430074, China; E-Mail: zhangys@wbgcas.cn (Y.S.Z.)

**Keywords:** *Panax ginseng*, ginsenoside, malonyl-ginsenoside Ra_3_

## Abstract

A new saponin, malonylginsenoside Ra_3_, was isolated from the fresh root of *Panax ginseng*, along with four known ginsenosides. The new compound was identified as (20*S*)-protopanaxadiol-3-*O*-(6-*O*-malonyl-*β*-D-glucopyranosyl(1→2)-*β*-D-glucopyranoside-20-*O*-*β*-D-xylopyranosyl(1→3)-*β*-D-glucopyranosyl(1→6)-*β*-D-glucopyranoside on the basis of extensive 1D and 2D NMR as well as HRESI-MS spectroscopic data analysis.

## 1. Introduction

**Figure 1:**
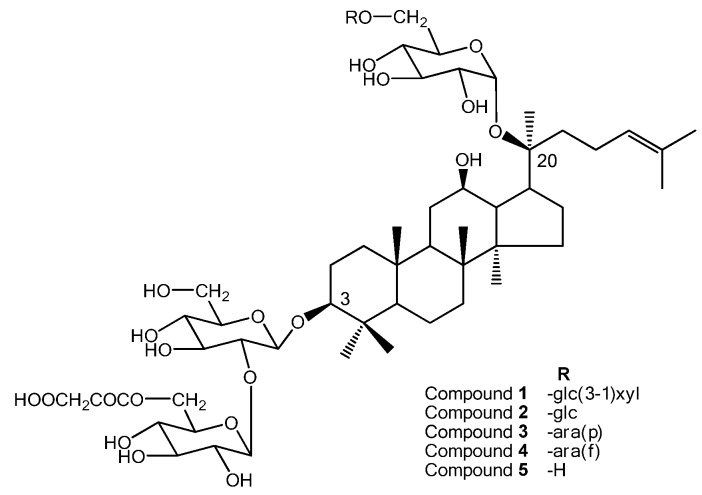
The structures of the isolated ginsenosides.

*Panax ginseng* C.A. Meyer has been used in China for thousands of years as a traditional medicine and proved to exhibit wide pharmacological properties, such as anti-fatigue, anti-diabetes, as well as activity in the prevention of cancer and the ageing process [1,2,3,4]. The major components contributing to its pharmacology activities were considered to be the ginsenosides, a group of steroidal saponins. Around 40 ginsenosides have been isolated and characterized till now, including the recent identified ginsenosides Ki and Km [5]. Among these known compounds, malonylginsenosides are natural ginsenosides that exist in both fresh and air-dried ginseng roots and which contain malonyl residues attached to the glucose units of the corresponding neutral ginsenosides [6]. Kitagawa *et al*. and Yamaguchi *et a*l. reported the presence of four acidic ginsenosides both in Asian and American ginseng [7,8]. Our previous pharmacology results showed that total malonyl-ginsenosides exhibit hypoglycemic effects on streptozotocin-induced diabetic mice [9]. During our continued studies on bioactive compounds from *Panax ginseng* [10,11,12], a novel ginsenoside, namely malonylginsenoside Ra_3_ (compound **1**), was isolated from a methanolic extraction of the fresh roots of *Panax ginseng*. This paper describes the isolation and structure determination of the new compound **1** (Figure 1). 

## 2. Results and Discussion

A crude methanolic extract of the fresh roots of *Panax ginseng* was subjected to open column chromatography on silica gel and then purified by preparative HPLC, to yield five ginsenosides, one of which, namely malonylginsenoside Ra_3_ (compound **1**), was new. The other four saponins were identified as known malonylginsenoside*-*Rb_1_ (compound **2**), malonylginsenoside*-*Rb_2_ (compound **3**), malonylginsenoside*-*Rc (compound **4**) and malonylginsenoside*-*Rd (compound **5**) by comparison of NMR data with those in the literature [6] and by comparison with authentic sample by ESI-MS, optical rotation and TLC.

### Characterization of compound ***1***

Compound **1** was obtained as a white amorphous powder and gave a peaks at *m/z* 1325.4 [M-H]^-^, 1281 [M-CO_2_]^-^, 1239 [M-COCH_2_COOH]^-^, 1107 [M-COCH_2_COOH-xyl]^-^, 945 [M-COCH_2_COOH-glc-xyl]^-^, 783 [M-COCH_2_COOH-xyl-2glc-H]^-^, 621 [M-COCH_2_COOH-xyl-3glc-H]^-^, 459 [M-COCH_2_COOH-xyl-4glc-H]^-^, in the negative ESI-MS, indicating its molecular weight to be 1326. The molecular formula was determined as C_62_H_102_O_30_ based on HRESI-MS [M+Na]^+^: *m/z* 1349.6348 [M+Na]^+^ (calcd. for C_62_H_102_NaO_30_, 1349.6353). IR (KBr) *ν*_max_/cm^-1^: 3423 cm^-1^ (OH), 1732 cm^-1^ (C=O), 1608 (C=C) and 1386 cm^-1^ (-CH_3_). Since compound **1** can’t be dissolved in pyridine-*d*_5_, we added 0.1 mL of D_2_O in 0.5 mL of pyridine-*d*_5_ as NMR solvent. Ginsenoside m-Rb_1_ (compound **2**) and the alkaline hydrolysis product of **1**, ginsenoside Ra_3_ (compound **1a**) were also dissolved in the same mixture solvent for NMR measurement. Analysis of the ^13^C-NMR spectrum (Table 1) and DEPT experiments, allowed the identification of eight methyl groups and six quaternary carbons. 

**Table 1:** The^ 13^C-NMR data of compounds **1, 2** and **1a**.^ a^

	1	2	1a		1	2	1a
C-1	40.0	40.1	39.3	3-Glu			
C-2	27.7	27.6	26.7	C-1’	105.5	105.8	105.0
C-3	90.9	90.7	89.0	C-2’	85.1	84.8	83.5
C-4	40.6	40.6	39.6	C-3’	78.5	78.5	78.0
C-5	57.4	57.4	56.5	C-4’	72.2	72.2	71.6
C-6	19.4	19.4	18.4	C-5’	78.5	78.8	78.0
C-7	36.0	36.0	35.1	C-6’	63.4	63.4	62.8
C-8	40.9	41	40.0	Glu			
C-9	51.0	51.1	50.1	C-1”	106.3	106.5	105.9
C-10	37.8	37.8	36.8	C-2”	77.4	77.5	77.1
C-11	31.2	31.3	30.9	C-3”	79.5	79.4	79.2
C-12	71.0	70.9	70.1	C-4”	71.6	71.7	71.6
C-13	50.0	50.2	49.3	C-5”	75.7	75.8	78.0
C-14	52.5	52.4	51.4	C-6”	66.1	66.1	62.8
C-15	31.8	31.9	30.9	20-Glu			
C-16	27.7	27.6	26.6	C-1’	98.7	98.8	98.1
C-17	52.8	52.8	51.7	C-2’	75.3	75.6	74.8
C-18	17.2	17.2	16.3	C-3’	78.5	78.8	78.0
C-19	16.9	16.9	16.0	C-4’	72.2	72.2	71.6
C-20	85.2	85.0	83.5	C-5’	77.4	77.2	77.1
C-21	23.3	23.3	22.7	C-6’	70.4	72	69.6
C-22	37.2	37.1	36.1	Glu			
C-23	24.3	24.2	23.3	C-1”	105.5	105.6	105.0
C-24	126.7	126.8	126.0	C-2”	74.7	75.6	74.2
C-25	132.8	132.6	130.8	C-3”	88.0	78.8	87.4
C-26	26.9	26.8	25.8	C-4”	71.7	72.2	71.3
C-27	19.0	19.0	17.9	C-5”	78.5	78.8	78.0
C-28	29.0	29.0	28.1	C-6”	63.0	63.4	62.4
C-29	17.5	17.5	16.5	Xyl			
C-30	18.3	18.3	17.3	C-1’’’	106.6		106.2
-O-CO	172.1	171.9		C-2’’’	75.7		75.2
CH_2_	41.9	41.9		C-3’’’	77.4		77.1
COOH	174.6	174.5		C-4’’’	71.2		70.8
				C-5’’’	67.8		67.2

^a^ Compounds **1**, **1a **and **2** were measured in C_5_N_5_-*d*_6 _(0.5 mL) plus D_2_O (0.1 mL).

**Figure 2:**
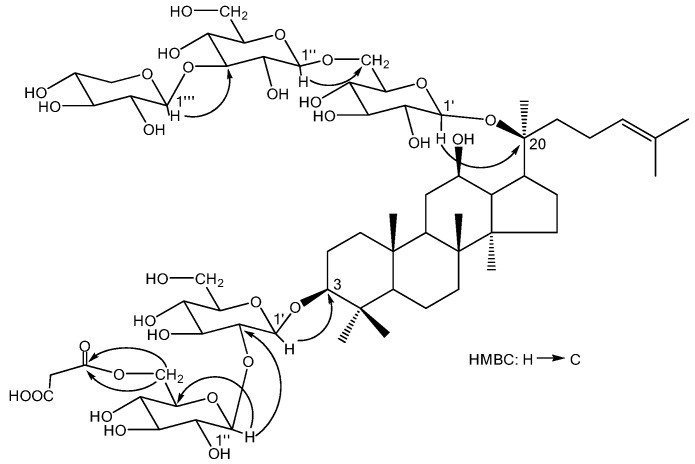
Partial HMBC correlation of compound **1**.

The ^1^H- and ^13^C-NMR spectroscopic data of compound **1** were similar to those of ginsenoside-Ra_3_ [13], except the data attributed to a malonyl group (*δ*_H_ 3.70, *δ*_C_ 172.1, *δ*_C_ 174.6). The malonyl group was assigned to C_3_-glc-C-6’’ position by HMBC experiment (Figure 2), which the protons of C_3_-glc-H-6’’ showed HMBC correlations with malonyl group (*δ*_C_ 172.1).

Malonyl group connection also caused a 2.7 ppm lower-field shift for C_3_-glc-C-6’’ (*δ*_C_ 66.1) than seen in ginsenoside-Ra_3_. Alkaline hydrolysis of compound **1** yield compound **1a**, which showed the structure identical to ginsenoside Ra_3_ by 1D NMR analysis (Table 1). The absolute configurations of the sugar moieties were further determined to be β-D-glucose and β-D-xylose by chiral GC analysis. The 20 position was determined as *S* conformation due to its similar NMR data with the known compounds **1a** and **2**. All the data above led us to identified the structure of **1** as (20*S*)-protopanaxadiol 3-*O*-(6-*O*-malonyl-*β*-D-glucopyranosyl(1→2)-*β*-D-glucopyranoside)-20-*O*-*β*-D-xylopyranosyl(1→3)-*β*-D-glucopyranosyl(1→6)-*β*-D-glucopyranoside, which we have named malonylginsenoside Ra_3_.

## 3. Experimental

### 3.1. General

The ^1^H- and ^13^C-NMR spectra were measured on a Bruker Avance DRX 500 NMR spectrometer, using TMS as an internal standard. Chemical shifts (*δ*) are expressed in parts per million (ppm), with the coupling constants (*J*) reported in Hertz (Hz). The ESI-MS spectra were recorded on a triple quadrupole mass spectrometer Quattro (VG Biotech, Altrincham, England) and the HRESI-MS spectra on a Bruker FT-ICRMS spectrometer. Column chromatographies were carried out with silica gel 60 M (200–300 mesh), Lichrospher RP-18 (20 μm); TLC was performed with silica gel plates (Macherey-Nagel, SilG/UV_254_, 0.20 mm), with spots detected by UV_254_ and H_2_SO_4_ (10%). HPLC were carried out with a Agilent 1100 system.

### 3.2. Plant material

The fresh root of *Panax ginseng* was collected from Fu-Song, Jilin, China, in August 2003, and identified by one of the authors, Prof. Yi-Nan Zheng. A voucher specimen (ZYC-RS-03-08) has been deposited in College of Chinese Medicinal Material, Jilin Agricultural University. 

### 3.3. Extraction and isolation

The root of *Panax ginseng* (10 kg) was extracted five times with MeOH-H_2_O (4:1), and the extract was concentrated under reduced pressure at 40 °C. The residue (~2 kg) obtained was suspended in water and subjected to D-101 resin column chromatography, using MeOH-H_2_O (0:1, 3:2) as eluted solvent to give total-ginsenoside (~300 g). The total-ginsenoside was applied to silica gel column and eluted with CHCl_3_-MeOH-H_2_O (6:4:1) to yield three fractions (F_1_-F_3_). Fraction F_1_ was further chromatographed on preparative HPLC eluted with gradient CH_3_CN-H_2_O (20% to 50%) to give the known saponins: malonylginsenoside*-*Rb_1_ (compound **2**, 100 mg), malonylginsenoside*-*Rb_2_ (compound **3**, 60 mg), malonylginsenoside*-*Rc (compound **4**, 65 mg), malonylginsenoside*-*Rd (compound **5**, 42 mg) and the new saponin malonylginsenoside Ra_3_ (compound **1**, 40 mg). Compound 1: ^1^H-NMR (400 MHz, 0.5 mL pyridine-*d*_5_ + 0.1 mL D_2_O, ppm): *δ* 0.73 (3H, *s*, H-19), 0.87 (3H, *s*, H-18), 0.95 (3H, *s*, H-30), 0.98 (3H, *s*, H-29), 1.17 (3H, *s*, H-28), 1.63 (3H, *s*, H-21), 1.65 (3H, *s*, H-26), 1.69 (3H, *s*, H-27), 5.29 (1H, *t-like*, H-24), 5.11 (1H, *d*, *J* = 7.2 Hz, C_20_-glc-H-1’), 4.93 (1H, *d*, *J* = 7.2 Hz, C_20_-glc-H-1’’) 4.85 (1H, *d*, *J* = 7.6 Hz, C_20_-xyl-H-1’’’), 4.81 (1H, *d*, *J* = 7.8 Hz, C_3_-glc-H-1’), 5.19 (1H, *d*, *J* = 7.6 Hz,C_3_-glc-H-1’’); ^13^C-NMR data, see Table 1.

### 3.4. Alkaline hydrolysis of compound ***1***

A solution of **1** (20 mg) in MeOH (3 mL) was treated with 5% KOH-MeOH (0.1 mL) and the whole mixture was stirred at room temperature (22 °C) for 30 min [6]. The reaction mixture was neutralized with cation exchange resin (SP20ss, Resindion S.R.L., Rome, Italy) and filtered. Removal of the solvent from the filtrate under reduced pressure gave a product which was purified by column chromatography with reversed-phase silica gel (Zorbax SB-C_18_) to furnish compound **1a**, which was determined to be identical with an authentic sample [6] by TLC comparison [CHCl_3_-MeOH-H_2_O (65:35:10, lower phase), *n*-BuOH-AcOH-H_2_O (4:1:5 upper phase)], IR(KBr), MS and ^13^C NMR spectral comparisons. Compound **1a**: IR (KBr) *ν*_max_ / cm^-1^: 3432, 1728, 1605, 1385, 1078; ESI-MS [-]: m/z = 1239 [M-H]^-^, 1107 [M-xyl]^-^, 945 [M-glc-xyl]^-^, 783 [M-xyl-2glc-H]^-^, 621 [M-xyl-3glc-H]^-^, 459 [M-xyl-4glc-H]^-^; ^1^H-NMR (400MHz, 0.5 mL pyridine-*d*_5_, ppm): *δ* 0.70 (3H, *s*, H-19), 0.84 (3H, *s*, H-18), 0.86 (3H, *s*, H-30), 0.96 (3H, *s*, H-29), 1.17 (3H, *s*, H-28), 1.49 (3H, *s*, H-21), 1.55 (3H, *s*, H-26), 1.58 (3H, *s*, H-27), 5.20 (1H, *t*, H-24), 5.04 (1H, *d*, *J* = 7.5 Hz, C_20_-glc-H-1’), 4.4.96 (1H, *d*, *J* = 7.6 Hz, C_20_-glc-H-1’’) 4.83 (1H, *d*, *J* = 7.6 Hz, C_20_-xyl-H-1’’’), 4.80 (1H, *d*, *J* = 7.8 Hz, C_3_-glc-H-1’), 5.26 (1H, *d*, *J* = 7.6 Hz, C_3_-glc-H-1’’); ^13^C-NMR data, see Table 1.

### 3.5. Acid hydrolysis of compound ***1***

To determine the stereochemistry of sugar moiety, compound **1** (2.0 mg) was refluxed with 6 N HCl (5 mL) at 100 °C for 2 h [14,15]. The mixture was extracted with CHCl_3_ to afford the aglycone, and the aqueous layer was neutralized with Na_2_CO_3_ and filtered. The aqueous layer was dried under vacuum and the residue was re-dissolved in H_2_O for sugar analysis by TLC with *n*-BuOH-AcOH-H_2_O (4:1:2) as the solvent. The sample spots were detected by spraying aniline hydrogen phthalate reagent (100 mL *n*-BuOH saturated by H_2_O, 0.96 g aniline and 1.66 g phthalic acid) and heating at 120 °C. D-Glucose and D-xylose were used as authentic standards. The absolute configuration of glucose was further determined by chiral GC analysis using a SatoChrom GC and a 0.25 mm × 25 m Hydrodexb-6-TBDM chiral capillary column (Macherey-Nagel, Germany). *β*-D-Glucose and *β*-D-xylose were used as an authentic GC standard. The aqueous layer residues mentioned above were re-suspended in dichloromethane (1 mL), and trifluoroacetic anhydride (50 µL) was added. The mixtures were allowed to react at room temperature overnight and dried under a stream of nitrogen at room temperature. The sugar derivatives were separated using the following temperature program: inlet temperature was set at 240 °C, with hydrogen carrier gas and a 1/20 split, using nitrogen makeup gas. Column temperatures started at 120 °C, ramped to 220 °C at 50 °C·min^–1^ and were maintained for 12 min.

## 4. Conclusion

A phytochemical investigation on the fresh root of *Panax ginseng* led to the isolation of a new saponin (20*S*)-protopanaxadiol 3-O-(6-O-malonyl-*β*-D-glucopyranosyl(1→2)-*β*-D-glucopyranoside)-20-O-*β*-D-xylopyranosyl(1→3)-*β*-D-glucopyranosyl(1→6)-*β*-D-glucopyranoside (**1**) along with four known ginsenosides (**2**–**5**).
